# Every Country, Every Family: Time to Act for Group B Streptococcal Disease Worldwide

**DOI:** 10.1093/cid/ciab859

**Published:** 2021-11-02

**Authors:** Joy E Lawn, Jaya Chandna, Proma Paul, Mark Jit, Caroline Trotter, Philipp Lambach, Ajoke Sobanjo Ter-Meulen

**Affiliations:** 1 Maternal, Adolescent, Reproductive & Child Health (MARCH) Centre, London School of Hygiene and Tropical Medicine, London, United Kingdom; 2 Department of Infectious Disease Epidemiology, Faculty of Epidemiology and Population Health, London School of Hygiene and Tropical Medicine, London, United Kingdom; 3 Department of Veterinary Medicine, University of Cambridge, Cambridge, United Kingdom; 4 Department of Immunization, Vaccines and Biologicals, World Health Organization, Geneva, Switzerlandand; 5 Bill & Melinda Gates Foundation, Seattle, Washington, USA

**Keywords:** Group B Streptococcus, maternal vaccines, neurodevelopmental impairment, vaccine readiness, vaccine market shaping

## Abstract

The global burden of Group B Streptococcus (GBS) was estimated for 2015 prompting inclusion of GBS as a priority in the Global Meningitis Roadmap. New estimates for the year 2020 and a WHO report analysing the full value of GBS maternal vaccines has been launched to advance evidence based decision making for multiple stakeholders. In this first of a 10-article supplement, we discuss the following (1) gaps in evidence and action, (2) new evidence in this supplement, and (3) what actions can be taken now and key research gaps ahead. We call for investment in the research pipeline, notably description, development, and delivery, in order to accelerate progress and address the large burden of GBS for every family in every country.

## GAPS IN EVIDENCE AND ACTION

Group B Streptococcus (GBS) is an important pathogen worldwide, yet still lacks concerted global attention and action. The Global Meningitis Roadmap highlights GBS as a priority, notably as the leading cause of meningitis in newborns and infants, adding urgency to GBS vaccine development and delivery [1]. Despite increasing interest in maternal GBS vaccines, evidence gaps have hindered a comprehensive assessment of the value of a vaccine. The papers in this supplement address priority data gaps and strengthen the evidence base to accelerate disease control plus development of a GBS vaccine.

Group B Streptococcus global burden estimates were published for the first time in a high-impact series in *Clinical Infectious Disease* in 2017 [2, 3], addressing some key misconceptions—notably, that GBS affected rich countries only. Indeed, GBS is an example of the inverse data law, where the highest burden occurs in countries and families with the least data [4]. More than 95% of the GBS burden is estimated to be in low- and middle-income countries (LMICs), with the highest incidence and mortality in sub-Saharan Africa, yet the least data inputs [3]. The health implications of GBS in pregnancy are not just for live-born neonates; it is an important cause of stillbirths [5] and also a risk factor for preterm births [6].

**Figure 1.**
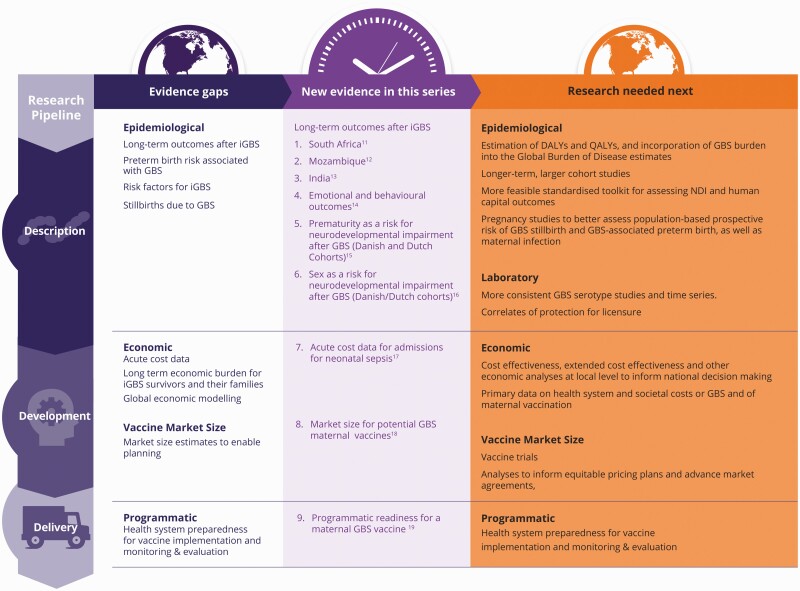
Evidence gaps: What is new in this series, and what’s next? Abbreviation: GBS, group B Streptococcus.

Evidence gaps were highlighted at that time, notably for epidemiological and economic data and analyses, as well as evidence foundational for planning programmatic and market roll-out (Figure 1). A top priority data gap was the dearth of data on long-term outcomes after invasive GBS in infancy. There were no cohort follow-up results beyond early childhood, no usable data from LMICs [7], and no data after GBS sepsis, which is more common than GBS meningitis. As a result, disability-adjusted life-years (DALYs) could not be calculated, nor were there any weights to calculate quality-adjusted life-years (QALYs), an increasingly popular alternative to DALYs, in LMICs. Furthermore, the potentially large burden of preterm birth associated with GBS could not be estimated. Primary data on both acute and long-term economic consequences after GBS were even more sparse, which impeded analyses of cost-effectiveness of interventions.

To reduce the burden of GBS, high-income countries have primarily relied on intrapartum antibiotic prophylaxis (IAP), with either risk-based screening or universal screening in the third trimester [8]. However, even with high coverage of IAP, this strategy is ineffective to reduce GBS-associated stillbirths, preterm birth, or late-onset GBS, and some cases of early-onset GBS still occur. Crucially, IAP is challenging in LMICs, which bear the highest burden of disease, and if all women colonized with GBS in pregnancy were given antibiotics, this would add millions of doses in an era with concern of rising antimicrobial resistance.

## NEW EVIDENCE TO INFORM ACTION

The first estimates of GBS burden [2, 3] raised awareness around the world, catalyzing the World Health Organization (WHO) to select GBS vaccines as their first in a new series of reports describing Full Value of Vaccines Assessments (FVVAs) [9]. Hence, the WHO GBS FVVA Time to Act Report [10] was developed, alongside this linked series of papers [11–19], presenting new data and analyses. In total, over 60 authors from 6 continents were involved in these 9 papers.

Several papers present the most comprehensive data so far on long-term outcomes reported from both LMICs and high-income countries (Figure 1). In 5 LMICs, children surviving GBS were followed up from age 18 months to 18 years with primary data on neurodevelopmental indicators (NDIs). Detailed results presented in this supplement identify increased odds of NDIs in invasive GBS (iGBS) survivors versus non-iGBS children in South Africa (odds ratio [OR]: 11.51; 95% confidence interval: 2.53–52.42) [11], Mozambique (OR: 8.41; 1.47–47.98) [12], and India (OR: 1.51; .65–3.46) [13], underlining the need for prevention and also for follow-up with care.

Emotional-behavioral problems can impact on the children, their families, and societal human capital, and may be challenging to measure consistently and across cultures. A paper by Chandna et al [14] reports primary data findings for 573 children using the Child Behavior Checklist (CBCL) across 5 LMIC sites. On average, we observed more total problems and more *Diagnostic and Statistical Manual of Mental Disorders, Fifth Edition* (DSM-V)–oriented problems for school-aged iGBS survivors compared with the non-iGBS group. At-risk neonates, including iGBS survivors, need long-term follow-up with integrated emotional-behavioral assessments and appropriate care. Scale-up will require simplified assessments that are free and culturally adaptable.

Two large national (Denmark and the Netherlands) electronic cohorts provide unique data after GBS in infancy into the second decade for more than 2000 iGBS children with matching 1 to 10 [15]. Here we report the first robust analyses of the interaction of GBS and preterm birth, with 36% (in Denmark) and 60% (in the Netherlands) of NDI risk in preterm children attributable to iGBS [16]. Another paper reports novel findings that boys with iGBS have an increased risk of NDI outcomes at the age of 5 years compared with girls with iGBS. Boys had a higher of risk of NDIs, with evidence for effect modification on an additive scale at the age of 5 years for any NDI (relative excess risk due to interaction between sex and iGBS = 1.28 [CI, −.53 to 3.09] in Denmark and 1.06 [CI, −5.12–7.25] in the Netherlands) [17]. Importantly, this multiplicative risk affects education and again underlines the need for longer-term follow-up.

Group B Streptococcus global burden estimates have been updated to year 2020, inclusive of these new data, using a Bayesian framework to integrate multiple data sources [10, 18]. Every country of the world has pregnant women colonized with GBS—approximately 20 million women globally. More than 390000 infants experience invasive GBS cases per year, resulting in 91000 (44000–187000) child deaths. In addition, there are 46000 (20000–111000) GBS stillbirths annually. Sub-Saharan Africa accounts for approximately 15% of the world’s population but about half of the burden of GBS cases and deaths. For the first time, we were able to estimate GBS-associated preterm births at 518000, albeit with wide uncertainty (36000–1142000). New input data on NDIs resulted in estimates of 40000 (14000–112000) survivors predicted to develop moderate and/or severe NDIs each year. Working with the Global Burden of Disease team, these new data will be used to calculate DALYs, permitting quantitative comparisons with other diseases.

The first primary data on the cost of admission for neonates with sepsis and/or meningitis are reported from 2 countries (South Africa and Mozambique) [19], noting that not a single paper was identified on this topic in a recent systematic review [20]. The substantial out-of-pocket costs identified will help inform further economic analyses.

Several vaccines are under development to address GBS disease. WHO has previously issued preferred product characteristics for a GBS vaccine to be used in pregnant women [21]. The first global cost-effectiveness analysis of GBS vaccination in the context of IAP is now available in the WHO GBS FVVA report [10]. This report recognizes the high disease burden and reports estimates of a substantial global impact of GBS vaccination, with potential as a feasible, sustainable, and cost-effective intervention if fairly priced.

The penultimate paper reports the first assessment of market size for GBS vaccines, considers the potential financial sustainability from a manufacturer’s perspective, and notes the importance of fair vaccine pricing to enable widespread demand [22]. The final paper in this collection reports on programmatic readiness from the perspective of policymakers and healthcare professionals, identifying, in particular, the need to appropriately package and present information to address stakeholder-evolving perceptions and promote well-informed decision making [23].

## TIME TO ACT

The GBS Full Value of Vaccines report led by WHO and the London School of Hygiene and Tropical Medicine was launched at the 2021 International Symposium on Streptococcus Agalactiae Disease, the only global conference on GBS. This event brings researchers, with implementers and vaccine developers, to accelerate action including regarding equitable introduction of forthcoming vaccines. Next-generation research is needed to address priority evidence gaps for GBS (Figure 1), but we must also enable next-generation researchers and implementers working in the highest-burden regions to lead advances in evidence and inform action.

## Notes

***Supplement sponsorship.*** This supplement is sponsored by Bill & Melinda Gates Foundation.

***Financial support.*** This work was supported by a grant (INV-009018) from the Bill & Melinda Gates Foundation to the London School of Hygiene & Tropical Medicine (Principal Investigator, J. E. L.) to the World Health Organization Immunization, Vaccines and Biologicals Department (INV-1175247).

***Potential conflicts of interest.*** All other authors report no potential conflicts. All authors have submitted the ICMJE Form for Disclosure of Potential Conflicts of Interest. Conflicts that the editors consider relevant to the content of the manuscript have been disclosed.
